# Venous Thromboembolism Prophylaxis in Hemophilic Patients Undergoing Total Hip or Knee Arthroplasty: Insights from a Single-Center Experience

**DOI:** 10.3390/medicina61040570

**Published:** 2025-03-22

**Authors:** Oana-Viola Badulescu, Paul-Dan Sirbu, Manuela Ciocoiu, Maria Cristina Vladeanu, Carmen Elena Plesoianu, Andrei Bojan, Dan Iliescu-Halitchi, Razvan Tudor, Bogdan Huzum, Mihnea-Theodor Sirbu, Norin Forna, Gheorghe Sofron, Wilhelm Friedl, Iris Bararu-Bojan

**Affiliations:** 1 Department of Pathophysiology, Morpho-Functional Sciences (II), Faculty of Medicine, University of Medicine and Pharmacy Grigore T. Popa, 700115 Iasi, Romania; oana.badulescu@umfiasi.ro (O.-V.B.); manuela.ciocoiu@umfiasi.ro (M.C.); maria.vladeanu@umfiasi.ro (M.C.V.); iris.bararu@umfiasi.ro (I.B.-B.); 2 Department of Orthopedics and Traumatology, Surgical Science (II), Faculty of Medicine, University of Medicine and Pharmacy Grigore T. Popa, 700115 Iasi, Romania; paul.sirbu@umfiasi.ro (P.-D.S.); rc_tudor@yahoo.com (R.T.); bogdan.huzum@umfiasi.ro (B.H.); mihnea-norin.forma@umfiasi.ro (N.F.); sfintugheorghesrl@yahoo.com (G.S.); 3 Department of Internal Medicine, Faculty of Medicine, University of Medicine and Pharmacy Grigore T. Popa, 700115 Iasi, Romania; carmen-elena.plesoianu@umfiasi.ro (C.E.P.); halitchi.iliescud@umfiasi.ro (D.I.-H.); 4 Department of Surgical Sciences, University of Medicine and Pharmacy Grigore T. Popa, 700115 Iași, Romania; andrei.bojan@umfiasi.ro; 5 Department of Othopedics and Traumatology, Wertheim Hospital, 97877 Wertheim, Germany

**Keywords:** hemophilia A, hemophilia B, thromboembolism, thromboprophylaxis

## Abstract

*Background and Objectives*: Total hip replacement and total knee arthroplasty carry a high risk of postoperative venous thromboembolism (VTE); therefore, anticoagulation prophylaxis is recommended in these patients. Unfortunately, there are no guidelines about VTE prophylaxis in patients with hemophilia who underwent these high-risk surgeries. To determine whether these patients have a high risk of VTE, we conducted a retrospective study on patients with hemophilia who underwent elective arthroplasty at our hospital in 2016. *Materials and Methods*: There were 11 patients with hemophilia A and B who underwent high-risk surgeries. Recombinant factor VIII or IX and also active recombinant Factor VII were used for perioperative hemostasis, and LMWH was administered for thromboembolic prophylaxis. Postoperatively, we collected information on the duration of factor VIII/IX infusion, VTE-prophylaxis, and complications. *Results*: Postoperative bleeding was minimal in most cases, with an average blood loss of 500 mL. No major thrombotic events were reported, and the need for transfusion was low, with only one patient requiring additional blood products. The VTE prophylaxis included prophylactic enoxaparin and hemostatic treatment. At the 1-year follow-up, we did not find any evidence of clinical VTE in our patients. *Conclusions*: Better risk stratification is needed to identify patients who would benefit from pharmacological prophylaxis. Total arthroplasty in hemophilic patients is feasible and safe when managed by a multidisciplinary team and supported by tailored antithrombotic prophylaxis protocols. The use of recombinant coagulation factors and LMWH ensures effective bleeding control and thromboembolic prevention, enhancing patient outcomes. These findings underscore the importance of individualized care in this high-risk population.

## 1. Introduction

Total hip and knee arthroplasty procedures have become increasingly common in the management of end-stage joint diseases, significantly improving patients’ quality of life. With advancements in medical care and an aging population, the number of arthroplasty procedures is expected to rise in the coming years. This trend is particularly relevant for hemophilic patients, who often develop severe joint damage due to recurrent hemarthrosis, necessitating surgical intervention. However, these patients present unique perioperative challenges, including an increased risk of both bleeding and thrombotic complications. As the demand for arthroplasty grows, optimizing thromboprophylaxis strategies while ensuring adequate hemostatic control will be crucial in minimizing complications and improving surgical outcomes in this high-risk population.

Between 45% and 84% of patients who have total hip replacements (THR) or total knee arthroplasty (TKA) will develop postoperative venous thromboembolic illness. A pulmonary embolism may also occur in 2% to 7% of these individuals, and 0.5% of them pass away as a result [1]. For these individuals, the American College of Chest Physicians strongly advises anticoagulation prophylaxis using fondaparinux, low-molecular-weight heparin (LMWH), or a vitamin K antagonist with a target international normalised ratio of roughly 2 to 3 [2].

The genetic X-linked bleeding disorders known as hemophilia are caused by a lack of either factor VIII (FVIII; hemophilia A) or factor IX (FIX; hemophilia B). Factor activity levels are correlated with disease severity; severe cases are classified as having less than 1% factor activity and frequently exhibit more frequent and substantial symptoms. Hemophilic patients may experience internal bleeding anywhere on their body; however, joints are the most frequently affected area (80%). Severe pain, deformity, decreased mobility, joint degradation, and the onset of crippling arthritis can result from this [2]. Consequently, hemophilic arthropathy can now be treated by elective joint arthroplasty. Benefits like enhancing function and mobility, lessening joint pain, and lowering the incidence of recurrent joint bleeding have all been shown [3]. The frequency of venous thromboembolism (VTE) in hemophilic patients is, however, poorly understood, which raises questions about the usefulness of anticoagulant prophylaxis following high-risk procedures [4].

The purpose of this study is to evaluate the safety and efficacy of pharmacological thromboprophylaxis in hemophilic patients undergoing total hip or knee arthroplasty. We hypothesize that with appropriate hemostatic management, anticoagulation can be safely administered to reduce the risk of venous thromboembolism without significantly increasing bleeding complications. Through this investigation, we aim to contribute valuable clinical insights that can help optimize perioperative care for this high-risk patient population.

## 2. Methods

### 2.1. Study Design

This retrospective study included hemophilic patients who underwent elective joint arthroplasty—either total knee arthroplasty (TKA) or total hip replacement (THR)—for hemophilic arthropathy at Sfântul Spiridon University Hospital in Iași, Romania, between 2016 and 2018. The study was approved by the institutional review board. All surgeries were performed under general anesthesia by an experienced orthopedic surgeon. Standard preoperative antibiotic prophylaxis was administered.

### 2.2. Inclusion and Exclusion Criteria

The study included 50 male patients with hemophilia, monitored at the Hematology Clinic of Sfântul Spiridon Hospital. Patients with hypersensitivity to coagulation factor components, disseminated intravascular coagulation, acute coronary ischemia, thrombosis, or embolism were excluded. Participants were divided into two groups: 45 patients with hemophilia A and 5 patients with hemophilia B. All patients were over 40 years old, as younger individuals with an indication for prosthetic surgery opted for continuous prophylactic substitution therapy and rehabilitation due to surgical risks. Pediatric hemophilic patients under 18 years old were treated at the Children’s Hospital and transferred to our center upon reaching adulthood.

Not all 50 patients proceeded with arthroplasty; some were included in the study to evaluate their bleeding predisposition. These patients formed a separate subgroup whose data were considered for assessing bleeding tendencies. In addition to categorizing patients based on hemophilia type, we also classified them according to disease severity (mild, moderate, or severe) to facilitate a more precise analysis of outcomes. This stratification allowed us to assess the impact of disease severity on surgical and postoperative complications.

### 2.3. Definitions

The severity of hemophilia is determined by the residual level of clotting factor present in the blood. Based on the serum levels of clotting factors FVIII or FIX, hemophilia is classified into three categories: mild, moderate, and severe. In the mild form, the clotting factor level ranges between 5 and 40% of the normal amount, while in the moderate form, it is reduced to 1–5% [5]. The severe form is characterized by clotting factor levels below 1% of the normal amount, posing a significantly higher risk for spontaneous and prolonged bleeding episodes.

The bleeding phenotype is characterized by a tendency for severe and prolonged bleeding, which may occur either spontaneously (without a specific triggering factor) or as a result of trauma or surgical interventions, depending on the severity of the clotting factor deficiency. These bleeding episodes primarily affect joints and muscles [6].

### 2.4. Coagulation Management

Preoperative treatment aimed to achieve target factor activity levels using recombinant factor therapy. Factor VIII (rFVIII) and Factor IX (rFIX) were administered every 8 h to maintain plasma levels of 80–100% until wound healing was achieved, followed by a gradual reduction over 10 days to sustain 30–60% activity. For patients with inhibitors, recombinant activated factor VII (rFVIIa) was given at an initial dose of 90 µg/kg preoperatively, then continued at 2–3 h intervals for 48 h, followed by 3–4 h intervals for 7 days and extended dosing up to 14 days. Tranexamic acid (25 mg/kg IV every 8 h) was administered as adjunctive therapy in patients with inhibitors to stabilize clot formation.

### 2.5. Thromboprophylaxis Protocol

Thromboprophylaxis was conducted following the American College of Chest Physicians (ACCP) guidelines, which recommend anticoagulation for up to 35 days after arthroplasty. However, in hemophilic patients, thromboprophylaxis was individualized to balance bleeding and clotting risks. Enoxaparin (60 mg SC once daily) was initiated 12–24 h postoperatively, once hemostasis was confirmed, and continued for two weeks. Anticoagulation was immediately discontinued if excessive bleeding or adverse reactions occurred. Hemoglobin levels and coagulation parameters were closely monitored throughout the postoperative period.

### 2.6. Prophylactic Antibiotics

Antibiotic prophylaxis was administered according to the orthopedic protocol, with cefuroxime 1.5 g every 8 h, starting 30 min preoperatively and continuing for three days postoperatively. In a hemophilia B patient with diabetes, the regimen was extended to seven days due to increased infection risk. Given the susceptibility of hemophilic patients to hematoma formation, delayed wound healing, and frequent venous access, prolonged antibiotic administration was justified to minimize infection risks, particularly periprosthetic joint infections (PJI).

### 2.7. Surgical Approach and Postoperative Management

Joint arthroplasty followed standard surgical techniques, with meticulous hemostasis to minimize intraoperative bleeding. Hemostatic agents were used as necessary, and excessive tissue trauma was avoided. Postoperatively, patients were monitored for bleeding, hematoma formation, and wound healing. Factor replacement therapy was continued until stable coagulation was achieved. Early mobilization was encouraged while balancing anticoagulation therapy to prevent thromboembolic events. Patients with comorbidities, such as diabetes, received extended antibiotic coverage to mitigate infection risks. Postoperative coagulation was also closely monitored, and fibrinogen levels were assessed following substitution therapy. In hemophilic patients with inhibitors receiving rFVIIa, fibrinogen levels were undetectable due to accelerated consumption via the extrinsic coagulation pathway. However, these patients did not exhibit signs of disseminated intravascular coagulation (DIC), as their postoperative bleeding was comparable to that of non-inhibitor patients. In contrast, fibrinogen levels remained stable in patients receiving Factor VIII or IX therapy, as normal thrombin generation was restored, ensuring efficient fibrin formation and clot stability. Thus, while factor administration does not directly modify fibrinogen levels, it affects its usage by restoring the coagulation pathway. In our study, patients undergoing joint replacement showed no platelet aggregation disorders. Factor VIII or IX administration does not directly affect platelet aggregation but restores thrombin generation, improving platelet activation. Factor VIII aids thrombin formation in hemophilia A, and Factor IX performs the same role in hemophilia B, restoring normal platelet function.

### 2.8. Assessment of Postoperative Complications

We aimed to assess postoperative complications, including bleeding, symptomatic VTE, and fibrinogen levels, as well as platelet aggregation disorders.

### 2.9. Statistical Analysis

Data were analyzed using SPSS 18.0. Collected variables included demographic and clinical characteristics, inhibitor presence, BMI, history of venous thromboembolism (VTE), HIV/hepatitis status, and postoperative complications. Correlations between coagulation parameters, bleeding tendencies, and surgical outcomes were evaluated.

In our study, patients undergoing joint replacement showed no platelet aggregation disorders. Factor VIII or IX administration does not directly affect platelet aggregation but restores thrombin generation, improving platelet activation.

## 3. Results

### 3.1. Demographics

**Figure 1:**
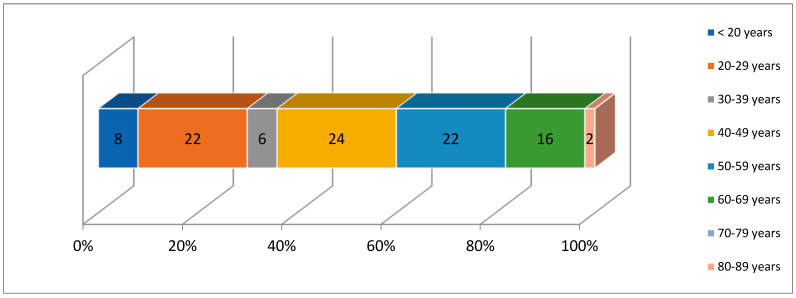
Age distribution.

The study included 50 male patients, with ages ranging from 18 to 80 years (mean age = 39.60 ± 14.58 years). A majority of patients (52%) were over the age of 40, with a higher percentage of hemophilia type A patients (53.3%) and hemophilia type B patients (40%) being in this age group (*p* = 0.571)—Figure 1. Regarding the bleeding phenotype, only 20% of patients with hemophilia type A and 20% of those with hemophilia type B exhibited bleeding phenotypes (*p* = 1.000).

### 3.2. Main Findings

The correlation between phenotype and hemophilia severity revealed the following (*p* = 0.798):

- Mild forms were observed in 2.5% of cases with a non-bleeding phenotype.
- Moderate hemophilia was recorded in 10% of patients with a bleeding phenotype and 10% of those with a non-bleeding phenotype.
- Severe forms of hemophilia were present in 90% of patients with a bleeding phenotype and 87.5% of those with a non-bleeding phenotype—Table 1.

The correlation between the affected joint and the phenotype revealed the following:

- The ankle was significantly more frequently associated with the bleeding phenotype (OR = 6.15; *p* = 0.014).
- The bleeding phenotype was observed with an odds ratio over three times higher in the knee (OR = 3.27; *p* = 0.134) and the hip (OR = 3.14; *p* = 0.144).

In patients with hemophilia type A, the number of affected joints was significantly higher in the presence of inhibitors (*p* = 0.001)—Table 2.

**Figure 2:**
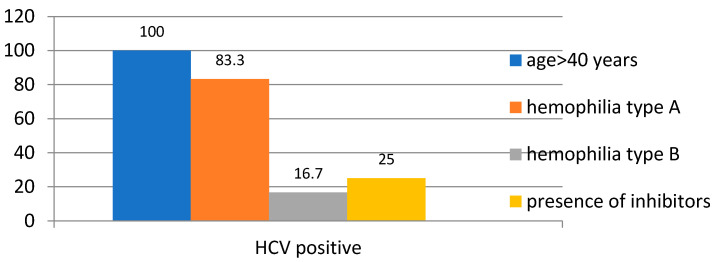
Distribution of Cases Based on HCV Positivity.

HCV was found to be positive in 24% of the patients, with all of them being over 40 years old (100%, *p* = 0.001). Additionally, 83.3% of patients with hemophilia type A tested positive for HCV (*p* = 0.05), and 25% of those with inhibitors present also tested positive for the virus (*p* = 0.023)—Figure 2.

### 3.3. Surgical Treatment and Subgroup Analysis

Surgical treatment was performed in 11 patients (22%), and 10 other patients (20%) were awaiting surgery. Currently, the 10 hemophilic patients are awaiting surgery, highlighting the complexity of these interventions, which demand a highly coordinated multidisciplinary team comprising hematologists, orthopedic surgeons, and intensive care specialists. Among those who underwent surgery, 25% of the patients with hemophilia A underwent surgery, while 20% of the patients with hemophilia type B operated. Additionally, 22.2% of patients with hemophilia type A and 60% of patients with hemophilia type B are currently awaiting surgical intervention (*p* = 0.048). When considering the presence of a bleeding phenotype, surgical treatment was recommended in 60% of cases with this phenotype, compared to 37.5% of cases with a non-bleeding phenotype (*p* = 0.095). The correlation between the affected joints and the need for surgery revealed the following findings:

- For the knee, 36.7% of cases underwent surgery, and 26.7% are currently awaiting surgery (*p* = 0.003).
- For the ankle, 33.3% of cases underwent surgery, and 33.3% are awaiting surgery (*p* = 0.012).
- For the hip, 45.5% of cases underwent surgery, and 27.3% are awaiting surgery (*p* = 0.049).
- For the elbow, 10.7% of cases underwent surgery, 14.3% are awaiting surgery, and 75% do not require surgery (*p* = 0.018).
- For the wrist, 25% of cases are awaiting surgery, and 75% do not require surgery (*p* = 0.043).

These results indicate that the need for surgical intervention varies by both joint and phenotype, with certain joints like the knee, ankle, and hip showing higher rates of surgery, while others like the elbow and wrist have lower surgical intervention rates. The presence of the bleeding phenotype also influences the likelihood of requiring surgery.

### 3.4. Postoperative Antithrombotic Treatment

Postoperative antithrombotic treatment was administered to 81.81% of the patients, meaning nine of the patients who underwent surgery, including the following specific groups:

- All of the patients were over the age of 40 (100%; *p* = 0.001).
- 88.9% of the patients with hemophilia type A (*p* = 0.05).
- 22.2% of the patients with hemophilia type A with inhibitors (*p* = 0.079).

These findings indicate that antithrombotic treatment was more commonly applied to patients over 40 years of age, reflecting a significant association between age and the use of postoperative anticoagulation therapy. Additionally, a higher percentage of patients with hemophilia type A received antithrombotic treatment compared to those with hemophilia type B, suggesting that the type of hemophilia may influence the decision for anticoagulation therapy. While the presence of inhibitors showed a trend toward association with increased antithrombotic treatment (*p* = 0.079), the relationship was not statistically significant. These results highlight the importance of age and hemophilia type in the management of postoperative anticoagulation in this patient population.

**Figure 3:**
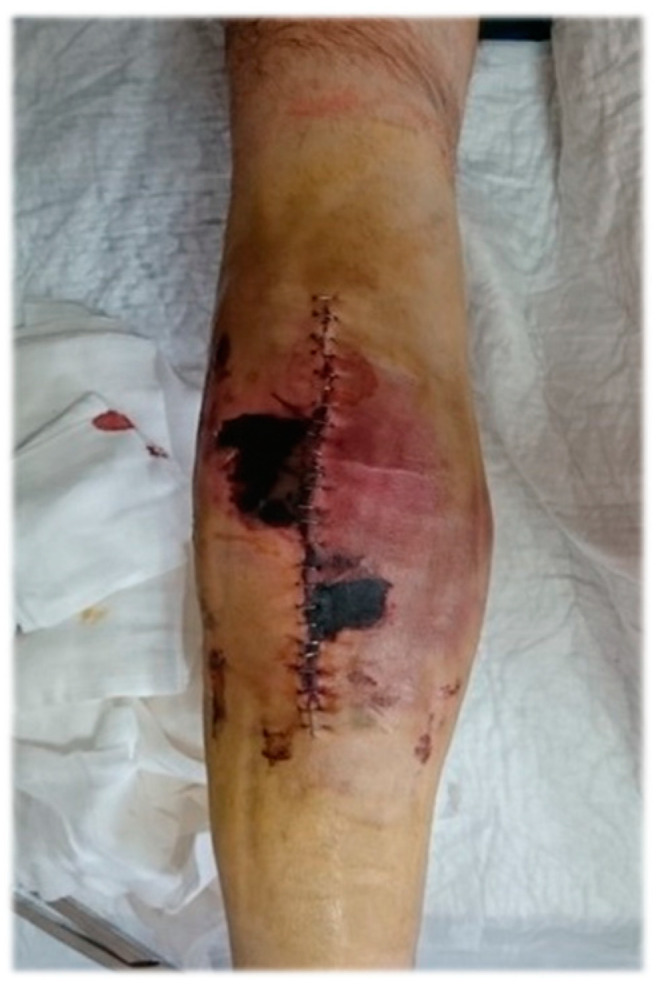
Skin necrosis after knee prosthesis surgery.

A patient diagnosed with severe hemophilia and the presence of inhibitors who underwent knee prosthesis surgery developed skin necrosis—Figure 3. This complication occurred because the patient had extremely thin skin, which made it more vulnerable to damage during the surgical process. The severity of hemophilia, coupled with the presence of inhibitors, contributed to this unusual postoperative complication.

One patient with moderate hemophilia (without inhibitors) who underwent knee prosthesis surgery experienced prolonged bleeding due to an undiagnosed factor VII deficiency. He received Novoseven for treatment, and low-molecular-weight heparin (Enoxaparin) was discontinued to avoid further bleeding risk.

A third patient with hemophilia type B also had comorbidities, including diabetes and hereditary thrombophilia. Testing positive for Factor V Leiden (homozygous), a genetic mutation increasing clot risk, was particularly relevant given the patient’s family history of recurrent spontaneous abortions. All hemophilic patients who underwent surgery received postoperative anticoagulation with enoxaparin, and no clinical VTE was observed at the 1-year follow-up.

### 3.5. Arthroplasties Performed in Patients with Severe Hemophilia and Present Inhibitors

Postoperative antithrombotic therapy was administered more frequently to patients over 40 years old, with hemophilia type A patients receiving higher rates of this treatment. This reflects a cautious approach to balancing clotting and thrombotic risks in older individuals and those with more severe disease profiles. Although the presence of inhibitors did not significantly impact the decision for antithrombotic therapy, the trend highlights the need for careful, individualized assessment in these complex cases.

Two patients with severe hemophilia A (Factor VIII < 1%) and present inhibitors were evaluated from an orthopedic perspective. The Bethesda titer of anti-FVIII antibodies was 9.3 units in the patient undergoing total hip arthroplasty and 9.6 units in the patient undergoing total knee arthroplasty. Both patients presented with decompensated hemophilic arthropathy, characterized by significant pain, functional impairment, and severe motor deficits, necessitating joint replacement surgery.

The procedures performed included a total hip arthroplasty on the left side for one patient and a total knee arthroplasty on the left side for the other, conducted by a multidisciplinary team (hematology, orthopedics, anesthesiology). Due to its short half-life of 2–3 h, rFVIIa was complemented with intravenous tranexamic acid at a dose of 25 mg/kg every 6–8 h, following established protocols. The combined use of rFVIIa and tranexamic acid was based on their synergistic effect, which enhances clot stability.

**Figure 4:**
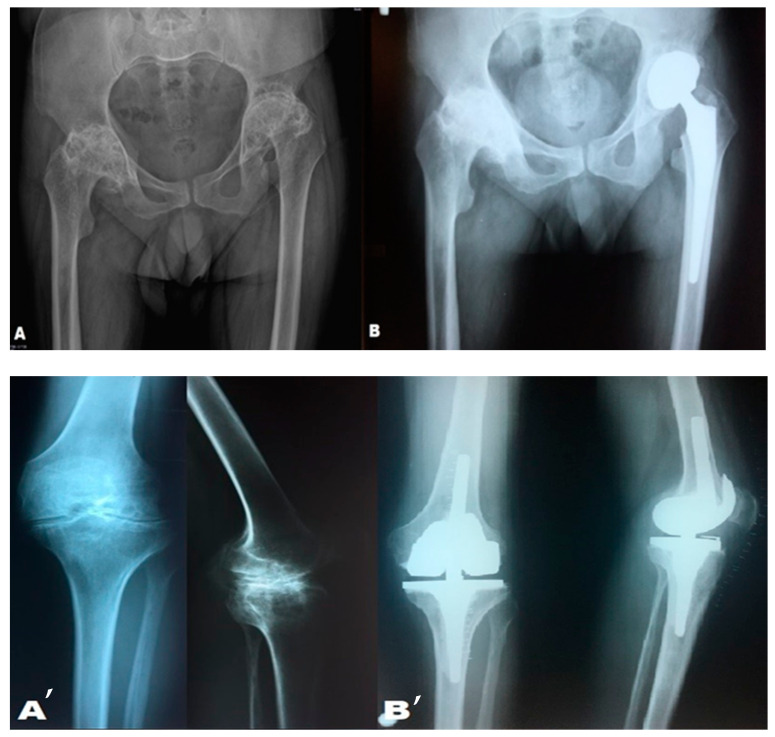
(**A**,**B**,**A’**,**B’**) X-rays of the pelvis and knee; anteroposterior and lateral views. Preoperative and postoperative X-rays (after prosthetic implantation) in patients with severe hemophilia and present inhibitors.

In the postoperative period, monitoring included hemoglobin and hematocrit levels, coagulation profile parameters, transfusion requirements, and potential orthopedic complications. Hemostasis was well-controlled, and the evolution regarding bleeding was excellent. Hemoglobin levels remained stable, with no transfusion required. Postoperative bleeding was minimal in both cases, approximately 450 mL, comparable to that of patients without hemophilia—Figure 4.

### 3.6. Arthroplasties Performed in Patients with Severe Type A Hemophilia

Eight patients with type A hemophilia, aged between 35 and 62 years, were evaluated from an orthopedic perspective. Among them, two had severe hemophilia (Factor VIII < 1%), and three had moderate hemophilia (Factor VIII = 1–5%). All patients presented with decompensated chronic knee arthropathies characterized by severe pain, functional impairment, and significant motor deficits. The surgeries performed included one total hip arthroplasty on the left side and four total knee arthroplasties on the left side, carried out by a multidisciplinary team (hematology, orthopedics, and anesthesiology). Recombinant Moroctocog alfa was used during the procedures. Postoperative evolution was excellent in most patients, with bleeding levels similar to those observed in individuals without hemophilia. However, one patient experienced prolonged bleeding due to an additional deficiency in coagulation Factor VII, necessitating the administration of one unit of red blood cell concentrate to address a drop in hemoglobin levels. Following the normalization of the coagulation profile with substitutive treatment using Moroctocog alfa, low-molecular-weight heparin (Enoxaparin) was administered to prevent thromboembolic complications. Notably, the severity of bleeding did not correlate with the residual Factor VIII level, and no significant differences in blood loss were observed between patients with severe and moderate hemophilia during the postoperative period. Patients aged 50 to 62 years showed postoperative outcomes comparable to those of younger patients who underwent joint replacements. These outcomes included significant pain reduction in the replaced joint, improved joint functionality, and a consequent increase in quality of life. Advanced age was not a barrier to performing these complex surgical interventions successfully.

### 3.7. Total Hip Arthroplasty Performed on a Patient with Moderate Type B Hemophilia and a Bleeding Phenotype

A 57-year-old patient with moderate type B hemophilia (Factor IX = 4%) presented with chronic left hip arthropathy due to frequent intra-articular hemorrhages and was seropositive for hepatitis C. The patient underwent left total hip arthroplasty after meeting the criteria for joint replacement. Perioperative treatment included recombinant Factor IX (Nonacog alfa) and postoperative thromboembolic prophylaxis with low-molecular-weight heparin. Despite his age, the patient had a favorable outcome, with significant pain relief, improved joint functionality, and enhanced quality of life. Postoperative bleeding was minimal, and no transfusion was needed. The hospital stay lasted 18 days, aligning with the average duration of 14–21 days. The average consumption of Factor IX during the perioperative period was approximately 120,000 units. Postoperative bleeding was around 550 mL, and the variations in hemoglobin levels from preoperative to postoperative were 14 g/dL to 9.1 g/dL, respectively.

## 4. Discussions

The most important results from the study indicate significant relationships between hemophilia type, joint involvement, and the need for surgical intervention. Patients over 40 years old accounted for the majority of cases, with a predominance of severe hemophilia in type A patients and moderate forms in type B. The ankle, knee, and hip joints were most frequently affected, with the bleeding phenotype strongly associated with ankle involvement. Surgical intervention was more common in patients with the bleeding phenotype, particularly for joints such as the knee, ankle, and hip. Notably, a higher proportion of patients with hemophilia type A underwent surgery compared to those with type B. Postoperative anticoagulation therapy was administered more frequently to older patients and those with hemophilia type A, suggesting that age and hemophilia type influence the management of thromboembolic risk. These findings emphasize the complex nature of managing hemophilic patients requiring joint replacement, with clear correlations between phenotype, joint involvement, and the need for surgery or postoperative treatment.

Since the underlying coagulation abnormality is thought to offer protection against VTE, orthopedic surgeons frequently steer clear of thromboprophylaxis. According to research by Krause et al., 32 hemophilia A individuals who had complete hip or knee replacement surgery without postoperative prophylaxis did not develop deep vein thrombosis (DVT). Similarly, when mechanical thromboprophylaxis or no prophylaxis was employed, additional trials have demonstrated a low incidence of postoperative VTE [6,7].

### 4.1. Summary of the Results

The age distribution of patients in this study highlights the predominance of individuals aged over 40 years, accounting for more than half of the cohort. While hemophilia type A and type B were observed in older patients, there was no significant correlation between advanced age and the risk of developing either type (RR = 1.06; 95% CI: 0.88–1.27). This finding suggests that while hemophilia is a lifelong condition, its type and onset are not influenced by the patient’s age at the time of diagnosis or assessment. These results also reflect the increasing life expectancy of patients with hemophilia due to advancements in medical management and supportive care.

### 4.2. Interpretation of the Results

Only 20% of patients exhibited a bleeding phenotype, regardless of hemophilia type, with no significant association between the two (*p* = 1.000, RR = 1.0; 95% CI: 0.79–1.26). This suggests other factors, such as genetic mutations, comorbidities, or treatment history, may influence bleeding presentation. Severity patterns varied significantly, with severe forms more common in type A (93.3%) than in type B (40%) (*p* = 0.007). Conversely, moderate forms were more frequent in type B (60%) than in type A (4.4%), likely due to genetic differences or responses to coagulation factor therapy. These findings emphasize the need for tailored management based on hemophilia type and severity. Most patients, regardless of phenotype, had severe hemophilia (90% of bleeding and 87.5% of non-bleeding phenotypes), while only 2.5% of non-bleeding cases were mild. This indicates that phenotype alone is not a reliable predictor of severity, reinforcing the need for comprehensive clinical and laboratory assessment. Our findings also highlight joint-specific bleeding risks. The ankle was significantly associated with a bleeding phenotype, while the knee and hip showed increased bleeding risk, though not statistically significant. These results suggest that joint characteristics and activity levels influence bleeding risk, underscoring the need for personalized joint management in hemophilia care.

The presence of inhibitors in patients with hemophilia type A was significantly correlated with a higher number of affected joints, illustrating the additional challenges inhibitors pose in managing hemophilic arthropathy. HCV positivity, observed in 24% of patients, was exclusively seen in those over 40 years old, with hemophilia type A and the presence of inhibitors being additional risk factors. This underscores the importance of screening and managing viral infections in older patients, particularly in those with complex hemophilia profiles [8,9].

Surgical interventions were more common in patients with hemophilia type A and those with a bleeding phenotype, particularly involving weight-bearing joints such as the knee and hip. The variation in surgical rates by joint highlights the functional and symptomatic impact of hemophilia-related joint damage. Interestingly, while elbow and wrist surgeries were less common, their outcomes suggest lower severity or different functional demands on these joints. The findings reinforce the importance of prioritizing surgical interventions based on both phenotype and joint-specific factors.

Complications from surgery were evident in specific cases, such as skin necrosis in a patient with severe hemophilia and inhibitors, where thin skin and disease severity posed additional challenges. Another case of prolonged bleeding due to an undiagnosed factor VII deficiency emphasizes the importance of comprehensive preoperative evaluation to mitigate risks. Moreover, the management of a patient with hemophilia type B and comorbid conditions like diabetes and hereditary thrombophilia underscores the importance of addressing overlapping genetic and systemic factors in hemophilia care.

Lastly, the wide use of anticoagulation therapy with Enoxaparine in all surgical patients and the absence of clinical venous thromboembolism (VTE) at the 1-year follow-up reflect effective postoperative management protocols. These findings underscore the importance of standardized, evidence-based practices in mitigating both bleeding and thrombotic risks in patients with hemophilia undergoing surgical interventions.

However, the risk of postoperative thromboembolic events in patients with hemophilia is generally considered low due to the inherent protective effects of reduced clotting factor activity, particularly in hemophilia A [10]. Unlike normal individuals, who experience a transient increase in FVIII levels postoperatively, hemophilia patients undergoing factor replacement therapy maintain FVIII levels at therapeutic but controlled levels, which are then tapered off. This controlled approach likely minimizes the hypercoagulable state observed in the general population post-surgery, reducing the risk of venous thromboembolism (VTE). However, the variability in thrombotic risk between hemophilia A and B, as well as in patients with comorbid conditions or congenital thrombophilic mutations, underscores the need for careful perioperative management tailored to individual risk profiles [11,12,13].

Studies have highlighted specific factors that may increase thrombotic risk in patients with bleeding disorders, particularly during major joint replacement surgeries. Risk factors include decreased joint mobility, obesity, rapid factor correction [14], and the presence of thrombophilic states [15], such as factor V Leiden mutation [16] or prothrombin gene mutations, which may paradoxically reduce bleeding tendencies in hemophilia patients [17,18]. These factors, along with the presence of HIV infection—a known independent risk factor for VTE—complicate the perioperative management of these patients. Additionally, Von Willebrand disease [19] has been associated with higher postoperative VTE risk, attributed to prolonged FVIII activity [20], raising questions about shared mechanisms in hemophilia patients receiving similar replacement therapies [21,22].

The variability in thromboprophylaxis approaches reflects ongoing uncertainty in managing VTE risk among hemophilia patients undergoing high-risk orthopedic procedures [23]. A survey of physicians in Hemophilia Treatment Centers revealed considerable differences in practice, with 67% supporting pharmacological anticoagulation prophylaxis and the rest relying on mechanical methods or no prophylaxis [23,24]. The choice of anticoagulant also varied, with options including enoxaparin, warfarin, and even aspirin, alongside mechanical interventions like compression stockings or sequential compression devices. This lack of consensus highlights the need for evidence-based guidelines to standardize care and balance the risks of bleeding and thrombosis in this unique population [25,26].

Given the complexities of managing hemophilia patients in the perioperative setting, individualized risk assessments remain essential. Factors such as the severity of hemophilia [27], the presence of inhibitors [28], comorbidities, and thrombophilic states should guide the decision-making process [29]. While pharmacological prophylaxis may be suitable for patients with controlled factor levels and higher VTE risk, mechanical prophylaxis may be preferred for those at greater bleeding risk [30,31]. Future studies are necessary to clarify the optimal approach to thromboprophylaxis, focusing on identifying which subsets of hemophilia patients may benefit most from these interventions while ensuring safety and efficacy.

### 4.3. Limitations of the Study

The study’s single-center design and relatively small sample size may limit the generalizability of the findings. Its retrospective nature introduces potential biases, such as selection bias and incomplete data recording, and the absence of a control group restricts comparative analyses. Variability in clinical management, short postoperative follow-up, and the presence of confounding factors, such as comorbidities, may affect the results. Additionally, the focus on Hemophilia A and B may not extend to other coagulopathies, and patient-reported outcomes, which provide valuable insights into quality of life, were not included. These factors should be considered when interpreting the study’s conclusions. Another limitation of our study is the unequal sample size between the two groups, which may introduce potential bias and affect the generalizability of the findings.

## 5. Conclusions

This study highlights the complexity of managing hemophilia patients of advanced age who are not significantly associated with hemophilia type, emphasizing the need for age-inclusive care. Bleeding phenotypes were not strongly linked to hemophilia type, suggesting that other factors may influence bleeding risk. The predominance of severe hemophilia in type A and moderate forms in type B underscores the need for tailored treatment approaches based on severity. The high prevalence of severe forms across phenotypes calls for personalized treatment models that consider age, phenotype, and severity to optimize outcomes.

The association of bleeding phenotypes with joints such as the ankle, knee, and hip highlights the importance of individualized joint management strategies. Surgical interventions, particularly for weight-bearing joints, were influenced by both phenotype and age, with successful outcomes linked to meticulous postoperative protocols, including anticoagulation therapy. Complications like skin necrosis and prolonged bleeding stress the importance of thorough preoperative evaluation and personalized perioperative management. These findings underline the need for a multidisciplinary approach to optimize outcomes, especially in complex cases involving inhibitors, viral infections, or genetic predispositions.

Given the complexities of managing hemophilia in the perioperative setting, individualized risk assessments are crucial. The severity of hemophilia, the presence of inhibitors, comorbidities, and thrombophilic states should guide decision-making. Pharmacological thromboprophylaxis may be appropriate for patients with controlled factor levels and higher VTE risk, while mechanical prophylaxis may be preferred for those at increased bleeding risk. Future studies are needed to clarify the optimal thromboprophylaxis approach, focusing on identifying the patient subsets most likely to benefit while ensuring safety and efficacy.

## Figures and Tables

**Table 1:** Correlation Between Phenotype and Hemophilia Severity. Form * Phenotype Crosstabulation.

Form	Phenotype (Bleeding)	Phenotype (Non-Bleeding)	Total
Severe	Count: 9	Count: 35	Count: 44
	% within Phenotype: 90.0%	% within Phenotype: 87.5%	% within Phenotype: 88.0%
Moderate	Count: 1	Count: 4	Count: 5
	% within Phenotype: 10.0%	% within Phenotype: 10.0%	% within Phenotype: 10.0%
Mild	Count: 0	Count: 1	Count: 1
	% within Phenotype: 0.0%	% within Phenotype: 2.5%	% within Phenotype: 2.0%
Total	Count: 10	Count: 40	Count: 50
	% within Phenotype: 100.0%	% within Phenotype: 100.0%	% within Phenotype: 100.0%

**Table 2:** Correlation Between the Number of Affected Joints, Type of Hemophilia, and the Presence of Inhibitors.

Hemophilia Type	Number of Joints	Inhibitors Present (Count)	Inhibitors Absent (Count)	Total
Type A	0	0 (0.0%)	1 (2.4%)	2.2%
	1	0 (0.0%)	20 (48.8%)	44.4%
	2	0 (0.0%)	10 (24.4%)	22.2%
	3	1 (25.0%)	8 (19.5%)	20.0%
	4	0 (0.0%)	2 (4.9%)	4.4%
	7	3 (75.0%)	0 (0.0%)	6.7%
Total		4(100%)	41 (100%)	100%
Type B	1	0 (0.0%)	3 (60.0%)	60.0%
	2	0 (0.0%)	2 (40.0%)	40.0%
Total		0 (100%)	5 (100%)	100%

## Data Availability

The original contributions presented in this study are included in the article. Further inquiries can be directed to the corresponding author.
